# Fully automated AI‐based quantification of tumour volume on PSMA PET‐CT images is associated with overall survival in prostate cancer

**DOI:** 10.1111/cpf.70104

**Published:** 2026-11-02

**Authors:** Johan Wittgren, Jacob Ingvar, Olof Enqvist, Måns Larsson, David Minarik, Lars Edenbrandt, Anders Bjartell, Elin Trägårdh

**Affiliations:** ^1^ Department of Translational Medicine Lund University Malmö Sweden; ^2^ Department of Clinical Physiology and Nuclear Medicine Skåne University Hospital Malmö Sweden; ^3^ Department of Urology Skåne University Hospital Malmö Sweden; ^4^ Chalmers University of Technology Gothenburg Sweden; ^5^ Eigenvision AB Lund Sweden; ^6^ Department of Radiation Physics Skåne University Hospital Malmö Sweden; ^7^ Department of Molecular and Clinical Medicine Gothenburg University Gothenburg Sweden

**Keywords:** [^18^F]‐PSMA‐1007, CNN, convolutional neural network, Cox regression, TLV, Tumour volume

## Abstract

**Introduction:**

Artificial intelligence (AI)‐based models can quantify tumour volume in positron emission tomography (PET) examinations using prostate specific membrane antigen (PSMA). It is, however, unknown whether this measurement of tumour volume contains prognostic information. The purpose of this study was to examine whether tumour volume measured by AI is associated with overall survival in patients with prostate cancer.

**Methods:**

Patients who had undergone [^18^F]PSMA‐1007 PET‐CT due to primary staging or biochemical recurrence (BCR) of prostate cancer at our centre were retrospectively included and followed for 5 years. Automatic quantification of tumour volume was performed using a previously developed fully automated AI model from our research group for PSMA PET‐CT analysis, without manual corrections. The tumour volume for the prostate, lymph nodes, and bone was calculated and summarized as Total Lesion Volume (TLV) and divided categorically (no detectable tumour, low, and high) as well as aggregated to whole‐body TLV and divided into tertiles (low, intermediate, and high). Mortality rates were compared using Cox‐regression and adjusted for age.

**Results:**

Among 267 patients in the final analysis, 48 (18%) died during follow‐up. Whole‐body TLV was significantly associated with mortality in the combined cohort (*p* = 0.001) and in the primary staging cohort (*p* = 0.014) but did not reach statistical significance in the BCR cohort (*p* = 0.061).

**Conclusions:**

Fully automated AI‐derived TLV from PSMA PET‐CT was associated with overall survival. These findings support the potential role of AI‐based tumour quantification as a potential prognostic biomarker in prostate cancer.

## 1 INTRODUCTION

Prostate cancer is among the most frequently diagnosed malignancies among men worldwide (Bray et al., 2024). Prognosis varies substantially between patients due to differences in tumour biology and disease stage, which in turn leads to a large variation in treatment options. Therefore, individualized treatment planning is crucial in prostate cancer management.

Positron emission tomography combined with computed tomography (PET‐CT) using prostate‐specific membrane antigen (PSMA) targeted radiopharmaceuticals has become an important imaging modality for diagnosis, staging, and treatment guidance in prostate cancer. PSMA is a transmembrane protein overexpressed in prostate cancer cells, making PSMA PET‐CT both sensitive and specific for detecting primary and metastatic disease in prostate cancer (Hope et al., 2019). PSMA PET‐CT has a higher accuracy compared to conventional imaging (Hofman et al., 2020; Perera et al., 2020).

Beyond lesion detection, PSMA PET‐CT provides quantitative information regarding tumour burden. Tumour burden measured on nuclear medicine images has long been recognized as a prognostic factor in prostate cancer. For example, Armstrong et al. demonstrated the importance of quantifying the tumour burden in bone scans (Armstrong et al., 2018). There have also been studies showing the prognostic information of tumour volume in bone using [^18^F]‐sodium fluoride PET (Lindgren Belal et al., 2017) as well as in lymph nodes using [^18^F]‐choline‐PET (Borrelli et al., 2021). In recent years, several studies have shown that tumour volume derived from PSMA PET‐CT is associated with clinical outcomes (Kleiburg et al., 2025; Küper et al., 2024; Seifert et al., 2020). More recently, the international PPP3 study with over 11,000 participants demonstrated that PSMA PET‐derived tumour burden, metastatic distribution, and PSMA expression provide powerful prognostic information across different stages of prostate cancer and outperform several established clinical risk classification systems. (Karpinski et al., 2026). We have recently shown that tumour volume holds prognostic value in patients undergoing PSMA PET‐CT as part of primary staging of prostate cancer or due to biochemical recurrence (BCR) (Ingvar et al., 2026).

However, quantification of tumour volume on PSMA PET‐CT has traditionally required manual or semi‐automated lesion delineation, which is time‐consuming and limits routine clinical implementation. To address this challenge, we recently developed and validated a fully automated artificial intelligence (AI)‐based model for the detection and quantification of tumour lesions in the prostate, lymph nodes and bone on PSMA PET‐CT images (Trägårdh et al., 2025). These methods dramatically reduce processing time, making assessment of tumour volume feasible in clinical settings.

The availability of a fully automated method creates an opportunity to integrate tumour burden assessment into clinical practice. However, before such implementation can be justified, it is necessary to establish whether the measurements generated by the AI model provide clinically meaningful prognostic information. Although a recent study by Zang et al. showed that automatically measured tumour volume could provide prognostic information in patients with metastatic castration resistant prostate cancer (mCRPC) receiving [^177^Lu]‐based therapy (Zang et al., 2025), evidence remains limited regarding patients in earlier stages of disease.

The aim of the present study was therefore to evaluate whether tumour volume measured entirely automatically by our previously validated AI model is associated with overall survival in patients undergoing [^18^F]PSMA‐1007 PET‐CT for primary staging or BCR of prostate cancer. We hypothesized that patients with a high AI‐measured tumour volume have a poorer overall survival compared to those with a low tumour volume.

## 2 METHODS

### 2.1 Study population

Patients referred for PET‐CT examination using [^18^F]PSMA‐1007 at Skåne University Hospital, Malmö and Lund, Sweden, between September 2019 and July 2021 were consecutively invited to participate and retrospectively evaluated. All participants provided written informed consent. Examinations were performed either for primary staging of newly diagnosed intermediate to high‐risk prostate cancer or due to BCR. Patient height, weight, and age at examination were recorded. D'Amico‐risk score was calculated based on prostate‐specific antigen (PSA)‐value, Gleason score, and clinical T‐stage, using referral notes and medical history.

### 2.2 Image acquisition

Patients were injected with 4 MBq/kg bodyweight (max 500 MBq) [^18^F]PSMA‐1007. Images were acquired 2 h after injection of the radiopharmaceutical using a Discovery MI PET‐CT (GE Healthcare). Scan time was 2 min/bed position (3 min if BMI ≥ 40). Images were acquired from the base of the skull to the mid‐thigh. Images were reconstructed using a block‐sequential regularization expectation maximization algorithm (Q.CLEAR, GE Healthcare) with a β‐value of 800. Time‐of‐flight, point spread function modelling, and a 256 × 256 matrix (pixel size 2.7 × 2.7 mm, slice thickness 2.8 mm) were used. A diagnostic CT with intravenous and oral contrast was performed of the examined area for attenuation correction and anatomical correlation.

### 2.3 Image analysis

All PET‐CT examinations were analyzed using a previously validated AI‐based model (Trägårdh et al., 2025), which has been shown to have a sensitivity similar to nuclear medicine physicians. None of the scans included in this study were part of the AI training dataset, ensuring independence of the evaluation. The AI algorithm automatically segmented suspected tumour lesions without any manual adjustments or corrections. The segmentation output directly included lesion labels for prostate/prostate bed, lymph nodes and bone. Volumetric measurements were subsequently derived from the segmentation masks for each lesion. For each patient, these measurements were aggregated to calculate the Total Lesion Volume (TLV) for each anatomical category as well as the whole‐body TLV, representing the overall tumour burden.

### 2.4 Statistical analysis

TLV was analyzed both for each anatomical site (prostate/prostate bed, lymph nodes, and bone) and for the whole body. For site‐specific analyses, patients were categorized into three groups: (1) no detectable tumour at the site, (2) low TLV (TLV ≤ median) and (3) high TLV (TLV > median). The low TLV group was used as the reference group. For whole‐body TLV, patients were divided evenly into tertiles (low, intermediate, and high), rather than using a ‘0 versus below/above median’ approach, as only six patients had a total TLV of zero. The lowest tertile was used as the reference group.

Mortality rates between the TLV‐categories were compared using a Cox proportional hazards model. Analysis was performed for the entire cohort, and sub‐analyses were performed separately for patients who had undergone examinations as part of primary staging or BCR. Hazard ratios were adjusted for age. Both adjusted and unadjusted results are reported in tables; in the text, only age‐adjusted numbers are reported unless otherwise specified. The overall significance of each TLV variable across categories was evaluated using likelihood‐ratio tests. Category‐specific effects are reported as hazard ratios (HRs) with 95% confidence intervals (CIs) relative to the specified reference category. Statistical significance was defined as *p*‐value < 0.05. All analyses were conducted using R, v 4.5.2.

### 2.5 Ethical approval

This study was approved by the local research ethics committee at Lund University (#2016/417, #2018/753, #2021–05734‐02, and 2022‐01302‐02) and followed the principles of the Declaration of Helsinki. All patients provided written informed consent.

## 3 RESULTS

### 3.1 Study population

A total of 267 patients were included in the study. The median age at the time of PET‐CT examination was 71 years. Of all examinations, 159 (60%) were performed for primary staging, and 108 (40%) for BCR. Detailed patient characteristics are presented in Table 1. The median PSA level for primary staging patients was 13 μg/L, and for patients with BCR 1.2 μg/L. In the primary staging cohort, a majority of patients (*n* = 118, 74%) were classified as high‐risk according to the D'Amico criteria, with the remainder primarily being classified as intermediate risk. In the BCR cohort, the majority of patients (*n* = 53, 49%) were defined as intermediate risk disease at the time of diagnosis. The median, min and max values of TLV for each patient group and anatomical location can be seen in Table 2.

**TABLE 1:** Patient characteristics.

Variable	*N* = 267
Dead	48 (18%)
Follow‐up time (years)	5.0 (0.1–5.3)
Age	71 (42‐87)
Subgroups	
BCR	108 (40%)
Primary staging	159 (60%)
D'amico score (primary staging)	
Low	1 (1%)
Intermediate	37 (23%)
High	118 (74%)
Missing	3 (2%)
D'amico score (BCR)	
Low	5 (5%)
Intermediate	53 (49%)
High	35 (32%)
Missing	15 (14%)
PSA (µg/L)	
Primary staging	13 (0.2–304)
BCR	IQR 7.6–22
	1.2 (0.1–97)
Missing	IQR 0.3–4.9
	6

*Note*: Continuous variables displayed as median (min‐max); categorical variables displayed as *n* (%). For PSA, interquartile range (IQR) is also noted.

Abbreviations: BCR, biochemical recurrence; TLV, Total lesion volume.

**TABLE 2:** Median value and quartiles of non‐zero TLV values separated by patient cohort and anatomical location. The number and percentage of patients with TLV > 0 and TLV = zero in each category is also displayed.

Cohort and Anatomical location	Median (IQR) TLV (cm^3^)	TLV > 0 (*n*, %)	TLV = 0 (*n*, %)
Total cohort			
Total	7.0 (3.1–17.9)	261 (98%)	6 (2%)
Prostate	4.9 (2.4–10.2)	196 (73%)	71 (27%)
Lymph node	1.3 (0.3–5.2)	185 (69%)	82 (31%)
Bone	2.1 (0.7–5.6)	160 (60%)	107 (40%)
Primary staging			
Total	9.1 (5.1–20.2)	159 (100%)	0 (0%)
Prostate	5.7 (3.7–11.5)	156 (98%)	3 (2%)
Lymph node	0.9 (0.3–3.6)	102 (64%)	57 (36%)
Bone	2.2 (0.8–6.0)	96 (60%)	63 (40%)
BCR			
Total	3.5 (1.3–10.2)	102 (94%)	6 (6%)
Prostate	1.4 (0.5–4.5)	40 (37%)	68 (63%)
Lymph node	1.6 (0.4–7.5)	83 (77%)	25 (23%)
Bone	1.9 (0.6–4.5)	64 (59%)	44 (41%)

*Note*: TLV values are displayed as median (IQR) among patients with TLV > 0.

Abbreviation: TLV, Total lesion volume.

### 3.2 Survival analysis

Among 267 patients included in the final analysis 48 patients (18%) died during follow‐up. Whole‐body TLV was significantly associated with overall survival in the entire cohort (*p* = 0.001). Compared with the low‐TLV reference group, patients with high whole‐body TLV had an HR of 3.20 (95% CI 1.50–6.80). Bone TLV was also significantly associated with overall survival (*p* = 0.016), with an HR of 2.20 (95% CI 1.07–4.54) for high versus low bone TLV. For prostate and lymph node TLV, the overall associations did not reach statistical significance (*p* = 0.063 and *p* = 0.071, respectively). However, the high‐TLV groups had higher mortality than the low‐TLV reference groups, with HRs of 2.23 (95% CI 1.10–4.54) for prostate TLV and 2.27 (95% CI 1.10–4.66) for lymph node TLV. The full results can be seen in Table 3 and the associated Kaplan–Meier graphs in Figure 1.

**TABLE 3:** Cox regression for overall survival based on tumour volume in the entire patient cohort, unadjusted and adjusted for age and divided by anatomical location.

Variable	Unadjusted		Adjusted	
	HR (95% CI)	*p*‐value	HR (95% CI)	*p*‐value
Whole body		<0.001		0.001
Low TLV	1 (Ref.)		1 (Ref.)	
Intermediate TLV	1.16 (0.48; 2.80)		1.19 (0.49; 2.86)	
High TLV	3.41 (1.61; 7.22)		3.20 (1.50; 6.80)	
Prostate		0.029		0.063
TLV = 0	1.39 (0.60; 3.21)		1.41 (0.61; 3.26)	
TLV ≤ median	1 (Ref.)		1 (Ref.)	
TLV > median	2.43 (1.20; 4.93)		2.23 (1.10; 4.54)	
Lymph nodes		0.043		0.071
TLV = 0	1.57 (0.71; 3.46)		1.59 (0.72; 3.50)	
TLV ≤ median	1 (Ref.)		1 (Ref.)	
TLV > median	2.42 (1.18; 4.96)		2.27 (1.10; 4.66)	
Bone		0.036		0.016
TLV = 0	1.00 (0.46; 2.18)		0.89 (0.41; 1.95)	
TLV ≤ median	1 (Ref.)		1 (Ref.)	
TLV > median	2.15 (1.04; 4.44)		2.20 (1.07; 4.54)	

*Note*: *P*‐values represent overall likelihood‐ratio tests for each TLV variable. HRs and 95% CIs represent category‐specific contrasts relative to the indicated reference group.

Abbreviation: TLV, total lesion volume.

**FIGURE 1:**
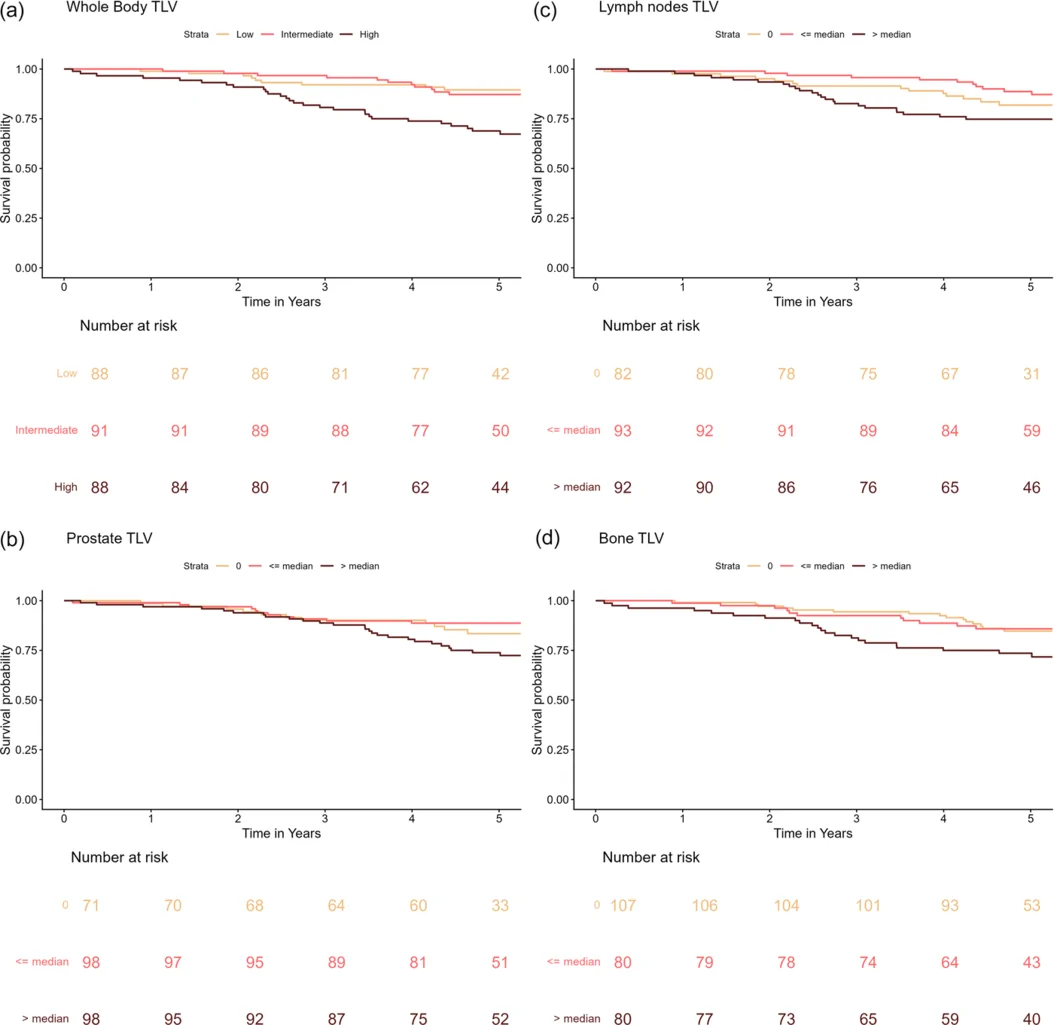
Kaplan–Meier curves of survival for the entire cohort for whole‐body TLV (top left), prostate TLV (bottom left), lymph node TLV (top right), and bone TLV (bottom right). Tumour volume divided into 0, ≤median, and >median for bone TLV, lymph node TLV, and prostate TLV. For whole bode TLV divided into tertiles (low, intermediate, and high). TLV, total lesion volume.

### 3.3 Primary staging and BCR cohorts

In the primary staging cohort, a total of 29 patients (18%) died during follow‐up. Whole‐body TLV was significantly associated with overall survival (*p* = 0.014). The HR for high versus low whole‐body TLV was 3.99 (95% CI 0.93–17.20). Bone TLV was also significantly associated with overall survival (*p* = 0.016), with an HR of 3.94 (95% CI 1.30–11.93) for high versus low bone TLV. The overall associations for prostate and lymph node TLV were not statistically significant (*p* = 0.091 and *p* = 0.180, respectively); the corresponding HRs for high versus low TLV were 2.42 (95% CI 0.98–6.01) and 2.34 (95% CI 0.92–5.96). All results from the staging cohort can be seen in Table 4.

**TABLE 4:** Cox regression for overall survival based on tumour volume in the patient cohort undergoing examination as part of primary staging, unadjusted and adjusted for age and divided by anatomical location.

Variable	Unadjusted		Adjusted	
	HR (95% CI)	*p*‐value	HR (95% CI)	*p*‐value
Whole body		0.003		0.014
Low TLV	1 (Ref.)		1 (Ref.)	
Intermediate TLV	1.39 (0.29; 6.71)		1.41 (0.29; 6.81)	
High TLV	4.60 (1.07; 19.69)		3.99 (0.93; 17.20)	
Prostate		0.048		0.091
TLV = 0	3.36 (0.40; 27.89)		4.37 (0.52; 36.81)	
TLV ≤ median	1 (Ref.)		1 (Ref.)	
TLV > median	2.79 (1.13; 6.88)		2.42 (0.98; 6.01)	
Lymph nodes		0.075		0.180
TLV = 0	1.41 (0.53; 3.80)		1.44 (0.53; 3.87)	
TLV ≤ median	1 (Ref.)		1 (Ref.)	
TLV > median	2.76 (1.10; 6.92)		2.34 (0.92; 5.96)	
Bone		0.031		0.016
TLV = 0	1.77 (0.55; 5.63)		1.63 (0.51; 5.19)	
TLV ≤ median	1 (Ref.)		1 (Ref.)	
TLV > median	3.63 (1.21; 10.95)		3.94 (1.30; 11.93)	

*Note*: *P*‐values represent overall likelihood‐ratio tests for each TLV variable. HRs and 95% CIs represent category‐specific contrasts relative to the indicated reference group.

Abbreviations: TLV, total lesion volume.

In the BCR patient cohort, a total of 19 patients (18%) died during follow‐up. The overall association between whole‐body TLV and overall survival did not reach statistical significance (*p* = 0.061), although the HR for high versus low whole‐body TLV was 3.47 (95% CI 1.26–9.61). No statistically significant overall associations were observed for prostate, lymph node, or bone TLV (*p* = 0.117, *p* = 0.401, and *p* = 0.249, respectively). The full results for the BCR subgroup are shown in Table 5.

**TABLE 5:** Cox regression for overall survival based on tumour volume in the patient cohort undergoing examination due to biochemical recurrence, unadjusted and adjusted for age, and divided by anatomical location.

Variable	Unadjusted		Adjusted	
	HR (95% CI)	*p*‐value	HR (95% CI)	*p*‐value
Whole body		0.068		0.061
Low TLV	1 (Ref.)		1 (Ref.)	
Intermediate TLV	1.47 (0.43; 5.01)		1.48 (0.43; 5.05)	
High TLV	3.38 (1.22; 9.33)		3.47 (1.26; 9.61)	
Prostate		0.111		0.117
TLV = 0	0.92 (0.31; 2.69)		0.93 (0.32; 2.73)	
TLV ≤ median	1 (Ref.)		1 (Ref.)	
TLV > median	3.67 (0.98; 13.67)		3.63 (0.97; 13.55)	
Lymph nodes		0.386		0.401
TLV = 0	1.95 (0.52; 7.25)		1.91 (0.51; 7.13)	
TLV ≤ median	1 (Ref.)		1 (Ref.)	
TLV > median	2.13 (0.67; 6.78)		2.10 (0.66; 6.70)	
Bone		0.326		0.249
TLV = 0	0.55 (0.18; 1.74)		0.50 (0.16; 1.60)	
TLV ≤ median	1 (Ref.)		1 (Ref.)	
TLV > median	1.28 (0.45; 3.64)		1.28 (0.45; 3.65)	

*Note*: *P*‐values represent overall likelihood‐ratio tests for each TLV variable. HRs and 95% CIs represent category‐specific contrasts relative to the indicated reference group. Abbreviation: TLV, total lesion volume.

## 4 DISCUSSION

In this study, AI‐measured tumour volume on [^18^F]PSMA‐1007 PET‐CT was associated with overall survival. Whole‐body TLV was significantly associated with overall survival and patients with a high whole body tumour burden in the overall cohort had a threefold increased risk of death compared with those with low tumour burden. This association persisted after adjustment for age. Site‐specific analyses showed a significant overall association between bone TLV and survival, whereas the overall associations for prostate and lymph node TLV did not reach statistical significance, despite higher mortality estimates in the high‐TLV groups. The pattern of associations differed between patients examined for primary staging and those evaluated for BCR, although no formal comparison between these cohorts was performed. Importantly, TLV was quantified entirely automatically without manual correction, demonstrating that prognostically relevant information can be extracted from PSMA PET‐CT examinations using AI. The fully automated approach makes large‐scale volumetric assessment technically feasible without additional manual segmentation and may ultimately support applications such as risk stratification and treatment monitoring, although further validation is required before clinical implementation.

Similar to previous fully automated tumour volume measurements (Zang et al., 2025), we also found a higher mortality with increasing tumour volume. Importantly though, Zang et al. studied mCRPC‐patients with very high tumour volumes and high mortality compared to the earlier stages of prostate cancer in our study, and so we believe these results contribute to the generalizability of the method.

Our results are also consistent with previous studies examining the effect of manually or semiautomatically acquired tumour volume on survival (Kleiburg et al., 2025; Küper et al., 2024; Seifert et al., 2020). Direct comparison with previous studies is problematic due to differences in patient population and methods, but they all tend toward increasing mortality when tumour volume increases.

Compared to our previous study using manual tumour delineation in a partially overlapping patient cohort (Ingvar et al., 2026), the present study demonstrates largely consistent findings using fully automated AI‐derived measurements. In both studies, increasing tumour burden was associated with poorer overall survival, with broadly similar patterns observed despite the different segmentation approaches. This suggests that prognostically relevant information from volumetric PSMA PET‐CT assessment can be obtained without the substantial time investment required for manual segmentation.

Both studies also demonstrated a seemingly paradoxical relationship where patients with small but quantifiable TLV in the prostate or lymph nodes tended to have a lower mortality compared to those with a measured TLV of zero. Since this phenomenon was observed using both manual and AI‐based segmentation methods, it is unlikely to be explained solely by limitations of the AI model. One possible explanation is selection bias related to the study population. All patients included in the study had known prostate cancer (newly diagnosed or BCR), so the absence of disease in the prostate may, in some cases, reflect a predominance of metastatic spread elsewhere, which is associated with a less favourable prognosis than localized disease. Another possibility is biological heterogeneity in PSMA expression. Approximately 5–10% of prostate cancers demonstrate low or absent PSMA expression (Minner et al., 2011), and a recent study suggested that absent intraprostatic PSMA expression may be associated with aggressive tumour subtypes, including intraductal carcinoma (Chen et al., 2025).

A separate consideration is the effect of false‐positive or non‐specific segmentations on the fully automated TLV measurements. The AI model used in this study has previously demonstrated good agreement with manual tumour volume measurements, although false‐positive lesions remain, particularly for lymph node and bone lesions (Trägårdh et al., 2025). For suspected bone metastases, the positive predictive value was approximately 70%, with 0.76 false‐positive lesions per patient, which was comparable to the inter‐reader agreement for manual assessment. However, unlike a clinical reading or study with manual tumour delineation, the present analysis used the complete AI output without physician review or removal of non‐specific segmentations. Consequently, even small false‐positive findings contributed to the calculated TLV. This introduces measurement noise which can lead to misclassification between TLV categories, particularly among patients with a low tumour burden. This could partially explain the small mortality difference between the low and intermediate whole‐body TLV groups.

In the present study, the AI model assigned a bone TLV > 0 to approximately 60% of patients in both the primary staging and BCR cohorts. In our previous study using manual delineation in a largely overlapping cohort, bone lesions were identified in approximately 20% of patients (Ingvar et al., 2026), suggesting that some small or non‐specific bone findings were included in the AI‐derived TLV. As noted above, even very small non‐specific findings will change the TLV categorization from TLV = 0 to TLV > 0. This is particularly relevant for [^18^F]PSMA‐1007, for which non‐specific bone uptake is a recognized interpretative challenge. Therefore, the AI‐derived bone TLV should not be interpreted as an exact measure of the true volume of bone metastases, and the risk of misclassification should be considered when interpreting the association between bone TLV and survival. Nevertheless, AI‐derived bone TLV remained significantly associated with increased mortality in the overall and primary staging analyses despite the absence of manual correction, indicating that the fully automated measurement retains prognostic information despite imperfect lesion classification. Whether removal of false‐positive lesions or expert correction would further improve prognostic performance should be evaluated in future studies.

The high TLV group was heterogeneous, with a wide range of measured volumes. Given this fact, combined with the risk of measurement noise at lower volume levels, alternative thresholds for TLV categorization might yield different associations. We deliberately used evenly distributed categories to avoid optimizing cut‐offs to the present dataset.

### 4.1 Limitations

Effect estimates generally indicated higher mortality with greater TLV, although the strength of statistical evidence varied across anatomical sites and patient subgroups, likely due to the limited number of events. We could have used a cohort with exclusively high‐risk cancers, but that would have limited the generalizability of the results. The retrospective single‐centre design may limit generalizability. Large prospective cohorts with longer follow‐up time will improve statistical power and allow more detailed analyses of additional TLV thresholds.

## 5 CONCLUSION

This study demonstrates that AI‐derived tumour volume from [^18^F]PSMA‐1007 PET‐CT is associated with overall survival in patients with intermediate and high‐risk prostate cancer, with the clearest associations observed for whole‐body and bone TLV. These findings suggest that automated volumetric analysis could provide prognostically relevant information without requiring additional physician time, supporting further evaluation of AI‐derived TLV as a potential imaging biomarker. Future research should validate these results in larger cohorts with longer follow‐up time, explore optimized volume thresholds, and assess integration of AI‐derived metrics into clinical decision‐making.

## CONFLICT OF INTEREST STATEMENT

Måns Larsson and Olof Enqvist are employees and stockholders of Eigenvision AB, a consultancy company working with research and development in automated image analysis, computer vision, and machine learning. Lars Edenbrandt serves on the board and is a stockholder of Slicevault AB, a company working with streamlining image management for biotech and pharmaceutical companies.

## Data Availability

Research data are not shared.
